# Functional regulation of syntaxin-1: An underlying mechanism mediating exocytosis in neuroendocrine cells

**DOI:** 10.3389/fendo.2023.1096365

**Published:** 2023-01-19

**Authors:** Xinquan Yang, Weifeng Tu, Xuzhu Gao, Qi Zhang, Jinping Guan, Junlong Zhang

**Affiliations:** ^1^ Anesthesia and Perioperative Medicine laboratory, the Affiliated Lianyungang Hospital of Jiangsu University, Lianyungang, China; ^2^ Faculty of Anesthesioloy, Suzhou Hospital Affiliated to Medical School of Nanjing University, Suzhou, China; ^3^ Department of Central Laboratory, Lianyungang Hospital Affiliated to Jiangsu University, Lianyungang, China

**Keywords:** synatxin-1, membrane fusion, exocytosis, SNARE, neuroendocrine cells

## Abstract

The fusion of the secretory vesicle with the plasma membrane requires the assembly of soluble N-ethylmaleimide-sensitive factor attachment protein receptor (SNARE) protein complexes formed by synaptobrevin, syntaxin-1, and SNAP-25. Within the pathway leading to exocytosis, the transitions between the “open” and “closed” conformations of syntaxin-1 function as a switch for the fusion of vesicles with the plasma membranes; rapid assembly and disassembly of syntaxin-1 clusters on the plasma membrane provide docking and fusion sites for secretory vesicles in neuroendocrine cells; and the fully zippered trans-SNARE complex, which requires the orderly, rapid and accurate binding of syntaxin-1 to other SNARE proteins, play key roles in triggering fusion. All of these reactions that affect exocytosis under physiological conditions are tightly regulated by multiple factors. Here, we review the current evidence for the involvement of syntaxin-1 in the mechanism of neuroendocrine cell exocytosis, discuss the roles of multiple factors such as proteins, lipids, protein kinases, drugs, and toxins in SNARE complex-mediated membrane fusion, and present an overview of syntaxin-1 mutation-associated diseases with a view to developing novel mechanistic therapeutic targets for the treatment of neuroendocrine disorders.

## 1 Introduction

Syntaxin family members vary in their cellular localization and tissue distribution, and all members of this family are involved in membrane fusion. Syntaxin-1 (STX1), a member of the syntaxin family is a nervous system-specific membrane protein responsible for fusion (1). In addition to neural tissues, syntaxin-1 is also expressed in non-neural tissues such as neuroendocrine (NE) cells, chromophobic granules in the adrenal medulla, and α, β, and δ cells in pancreatic islets. Two isoforms of syntaxin-1, syntaxin-1A and syntaxin-1B, show significant homology at the amino acid level as well as in the SNARE motif (2). Moreover, both isoforms of syntaxin-1 are members of the group of SNAREs (soluble N-ethylmaleimide sensitive factor attachment protein receptors), which are core components of the exocytosis machinery. During exocytosis, syntaxin-1, together with accessory proteins, regulate calcium-triggered fast membrane fusion. This process is also affected by multiple factors such as accessory proteins, lipids, protein kinases, toxins, drugs and genetic mutations. Although SNAP-25 and synaptobrevin (VAMP) can arguably be considered as members of SNAREs, they are outside the scope of this review and are covered in depth elsewhere.

This review focuses on the mechanisms and factors that regulate the functions of syntaxin-1, with an emphasis on the key regulatory pathways of SNARE assembly, such as the transitions in the conformations of syntaxin-1, syntaxin-1 clusters, and precise assembly process.

## 2 Physiological function and structure of syntaxin-1

### 2.1 Structure of syntaxin-1

**Figure 1:**
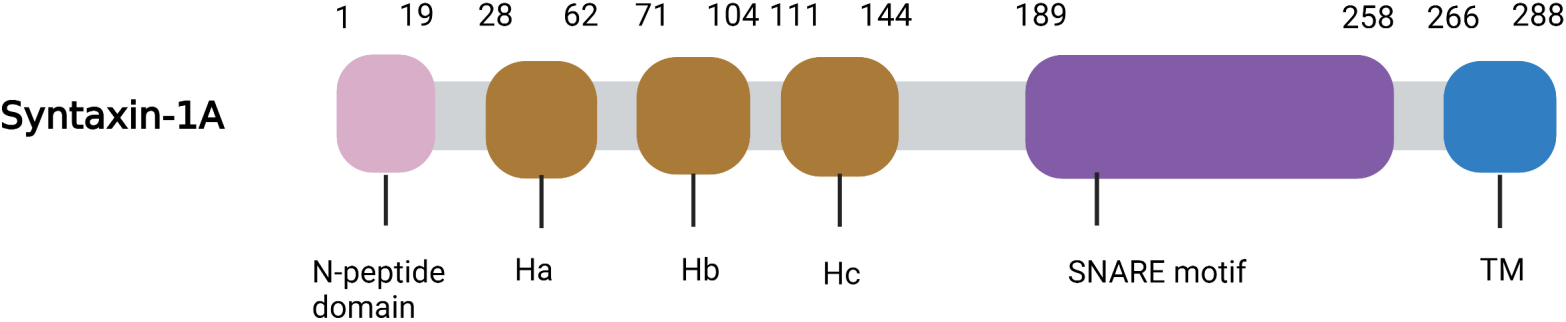
Ribbon diagrams of Syntaxin-1A. The N-peptide domain is shown in Pink, the Habc domain is shown in brown, the SNARE motif in purple and the TM in blue.

Syntaxin-1 is a helical protein with multiple domains that is localized on the plasma membrane. Syntaxin-1 consists of a short N-peptide (aa 1-9 or 1-28), the Habc domain (aa 29-144, formed by three helices, Ha, Hb, and Hc), the linker region (aa 156-187), followed by the SNARE motif (aa 189-259, H3 helix), a juxtamembrane region (aa 258-270), and a C-terminal transmembrane region (TMR; aa 266-288) (3) (**Figure 1**). The N-peptide at the N-terminus is a peptide composed of 20 amino acids, and it plays an essential role in exocytosis (4). The regulatory domain—the Habc domain—is highly conserved in the mammalian system, is located adjacent to the N-peptide, and can bind with Munc-18-1 (5, 6). The linker region between Habc and H3, residues 156-187, is essential for the syntaxin-1 conformational switch (6). The SNARE domain (H3) is a key binding site for syntaxin-1 to form a four-helix bundle with SNAP-25 and synaptobrevin (VAMP) for membrane fusion. The juxtamembrane region is highly conserved (7) and can interact with phosphoinositides (e.g., PIP3 and PIP2). And this interaction is achieved through the formation of salt bridges (specific electrostatic interactions) between the positively charged residues in the juxtamembrane region and the negatively charged phosphate groups of lipids in the plasma membrane, which can recruit syntaxin-1 to the fusion site. In addition, the transmembrane domain (TMR) is a C-terminal hydrophobic stretch with poor sequence conservation that potentially functions as a membrane anchor (8, 9).

### 2.2 Physiological functions of syntaxin-1

One of the most extensively characterized functions of syntaxin-1 is the assembly of the SNARE complex, which triggers exocytosis. Exocytosis occurs through the rapid and regulated fusion of secretory vesicles with the plasma membrane, and this fusion is driven by the assembly of soluble N-ethylmaleimide-sensitive factor (NSF) attachment protein (SNAP) receptor (SNARE) protein complexes (10). Many studies that have contributed to our understanding of exocytosis in different cell types placed a significant focus on synaptic release and neurotransmission. This process involves the vesicle cycle, which is the basis for neuronal communication and information processing in the brain.

The vesicle cycle can be subdivided into distinct steps, including vesicle mobilization, docking, priming, membrane fusion, and recycling (11). In brief, once an action potential reaches the presynaptic membrane, it opens calcium channels that trigger an inflow of calcium ions into the presynaptic membrane, and the accumulated neurotransmitter synaptic vesicles then fuse with the plasma membrane, releasing their contents into the synaptic cleft. Priming is a general term assigned to events that occur prior to Ca^2+^-dependent membrane fusion, which are likely to include preparation of the secretory vesicle and presynaptic membrane prior to vesicle docking. The critical events are listed below: The SNARE complex is disassembled by the joint action of α-SNAP and NSF with Mg-ATP (12, 13). The dissociation of Munc-18 from syntaxin-1, which is regulated by tomosyn or Munc13-1, provides space for the next step of syntaxin-1 combination (12). Syntaxin-1 and SNAP-25 form a dimeric t-SNARE, which then forms a ternary SNARE complex with v-SNARE (also known as synaptobrevin, VAMP) on the opposing membrane (13, 14). Synaptotagmin-1 and complexin bind to trans-SNARE complexes (15). Membrane fusion refers to the events that are triggered by the binding of Ca^2+^ and Ca^2+^ sensors, which transmit signals to the SNARE complex that allow the fusion of the presynaptic membrane and vesicles and the release of neurotransmitters (13, 15). Synaptic transmission involves a fast synchronous phase and a slower asynchronous phase of neurotransmitter release that are regulated by distinct Ca^2+^ sensors (16). During an action potential, Ca^2+^ entering a presynaptic terminal and binding to synaptotagmin (a Ca^2+^ sensor) triggers release by stimulating synaptotagmin binding to a core fusion machinery composed of SNARE and SM proteins that mediate membrane fusion during exocytosis (17). Complexin adaptor proteins assist synaptotagmin by activating and clamping this core fusion machinery. Synaptic vesicles containing synaptotagmin are positioned at the site of vesicle fusion by a protein complex containing RIM proteins. The RIM proteins activate docking and priming of vesicles and simultaneously recruit Ca^2+^ channels to active zones, thereby connecting the primed vesicles to Ca^2+^ channels in a single complex. This architecture allows direct flow of Ca^2+^ ions from Ca^2+^ channels to synaptotagmin, mediating tight millisecond coupling of an action potential to neurotransmitter release (fast synchronous release) (17). In contrast, Doc2 is a Ca^2+^ sensor that is kinetically tuned to regulate asynchronous neurotransmitter release (16). During membrane fusion, trans-SNAREs are converted to cis-SNAREs, which can be disassembled by the joint action of α-SNAP and NSF with Mg-ATP. In addition, the ternary SNARE complex is called the trans-SNARE complex when syntaxin-1 and VAMP reside in different membranes that can bring the vesicle closer to the plasma membrane. In contrast, cis-SNARE is the fully assembled ternary SNARE complex with both the ayntaxin-1 and VAMP residing in the same membrane, which occurs simultaneously with membrane fusion and synaptic release (12, 13).

Among other functions, syntaxin-1 is believed to interact with the “synprint” site of the calcium channels, which regulates N- and P/Q-type channels (18). Most synaptic P/Q- and N-type calcium channels work in conjunction. Calcium channels are essential to control the release of neurotransmitters, and they are covered in depth elsewhere.

## 3 Regulation of accessory proteins in key events of syntaxin-1

### 3.1 Regulation of accessory proteins in syntaxin-1 clustering at the plasma membrane

Syntaxin-1 constantly switches between the freely diffusing (mobile) and clustered (immobile) states at the plasma membrane, which serves as a mechanism for the rapid assembly and disassembly of syntaxin-1 nanoclusters in response to the transformation of syntaxin-1 mobility at the plasma membrane. Syntaxin-1 clusters in NE cells are functional docking and fusion sites for exocytic vesicles, which cluster and diffuse at these sites (19). Syntaxin-1 nanoclusters also contain pre-assembled SNARE complexes. Therefore, upon transmitter release, the SNARE complexes disassemble, and syntaxin-1 is released from the nanoclusters (20).

Syntaxin-1 is highly mobile at the plasma membrane, which allows the formation of clusters of syntaxin-1. The interaction between syntaxin-1 and other SNARE proteins affects the mobility of syntaxin-1 at the plasma membrane. Recent research has shown that syntaxin-1 is present more frequently at the synaptic site, and the mobility of syntaxin-1 at the synaptic region is significantly reduced. In addition, interference with syntaxin-1 and the interactions between syntaxin-1 and other SNARE proteins could alter the frequency of movement and dwelling of single syntaxin-1 molecules (21). One study also found that H3 domain deletion of syntaxin-1 and enzymatic cleavage of SNAP-25 would reduce the affinity between syntaxin-1 and SNAP-25, resulting in increased mobility and reduced frequency of dwelling of syntaxin-1 (22). Tetanus toxin (cleaves VAMP) prevented the formation of the SNARE complex, leading to an increase in the mobility of syntaxin-1 and a reduction in the size of the syntaxin-1 nanocluster on neuromuscular junction (NMJ). Moreover, blocking the dissociation of the SNARE complex also reduced the mobility of syntaxin-1 (21).

The domain 3a hinge-loop of Munc18-1 controls the conformational change of syntaxin-1 and the mobility of syntaxin-1 in the presynaptic membrane. Studies have shown that the expression of Munc18-1 with the domain 3a hinge-loop defects can eliminate the decrease in the mobility of Syntaxin-1 caused by wild-type Munc18-1 under certain stimulation. Further studies showed that botulinum toxin e (hydrolyzed SNAP-25) also eliminated the inhibition of the mobility of syntaxin-1 by Munc18-1 (23). These findings indicate that the Munc18-1-mediated reduction in syntaxin-1 mobility may be caused by the assembly of the SNARE complex.

Weak homogeneous interactions between syntaxin-1 molecules allow syntaxin-1 to cluster at the plasma membrane and maintain a dynamic equilibrium between the free and clustered subgroups of syntaxin-1 molecules (24). Notably, the transmembrane domain (TMR) of syntaxin-1 can form clusters on the plasma membrane alone, promoting the loose cluster of Syntaxin-1. Moreover, the interaction with the SNARE domain traps syntaxin-1 molecules into nanoclusters (25), promoting tight clustering of the molecules within the clusters.

### 3.2 Regulation of accessory proteins in conformational conversion of syntaxin-1

**Figure 2:**
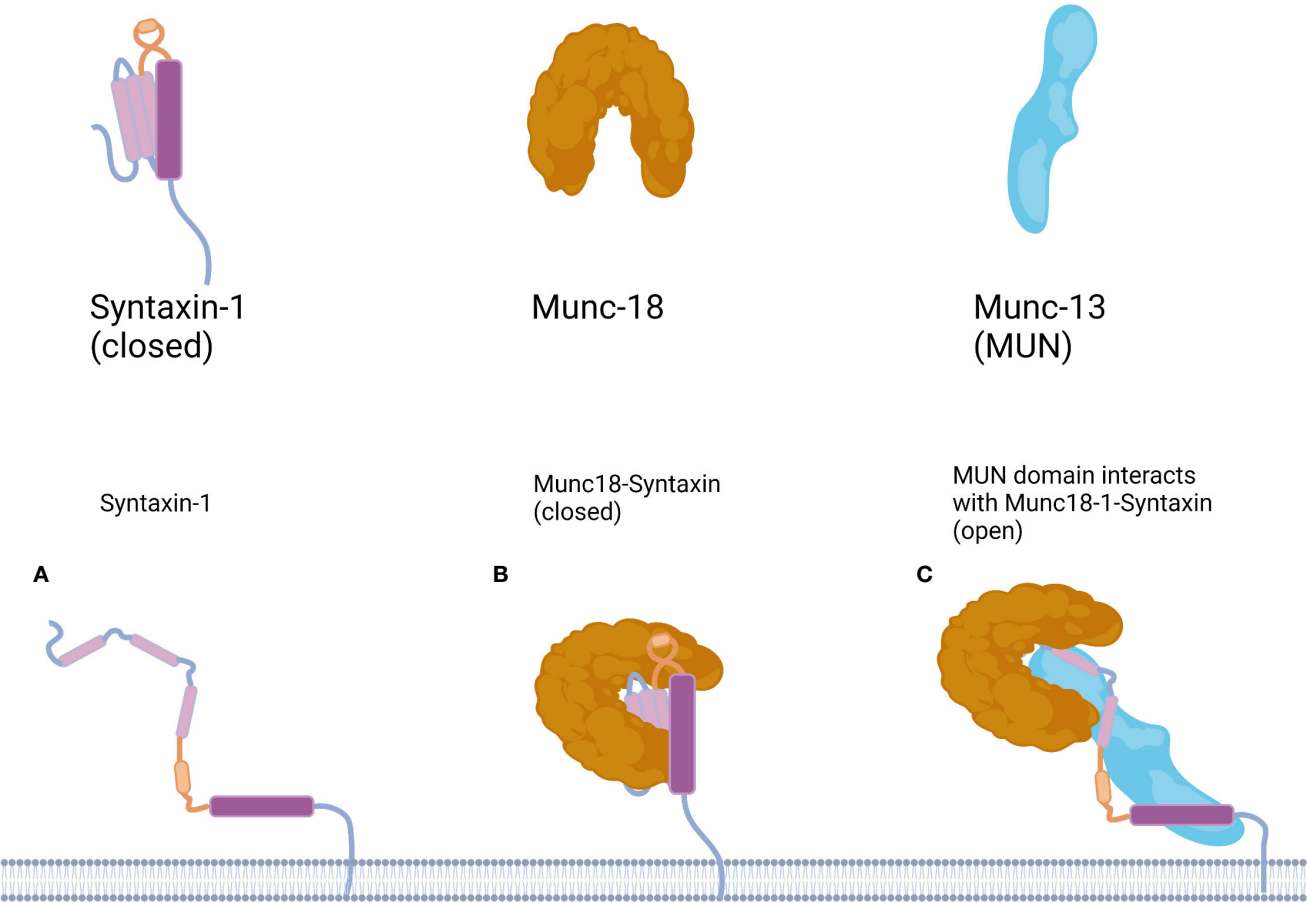
Working model for the mechanism underlying the conformational transition of Syntaxin-1. **(A)** In the Syntaxin-1 molecule the Habc domain is shown in pink, the linker region in yellow and the SNARE motif in purple. **(B)** Munc18-1 protects Syntaxin-1 into a closed conformation, in the open conformation, the Habc domain of Syntaxin-1 folds back into the SNARE domain to form a closed conformation. **(C)** Binding of the MUN domain to Munc18-1 and Syntaxin-1 is proposed to transiently open Syntaxin-1. Munc18-1/syntaxin-1/MUN complex leads to a conformational change in the syntaxin-1 linker region may expose the N-terminal end of the H3 domain of syntaxin-1, thereby providing a nucleation site for SNAP-25 and synaptobrevin-2 binding, and in the open conformation the Habc domain is released from the H3 domain.

Most syntaxin members, including syntaxin-1, can adopt a non-activating “closed” conformation. When syntaxin-1 is not involved in the assembly of the SNARE complex, the Habc domain folds back into the SNARE domain, forming a closed conformation. At this point, molecular interactions between the Habc and SNARE domains prevent the SNARE domain from interacting with other proteins. In the open conformation, the Habc domain is released from the H3 domain, facilitating the interaction of syntaxin-1 with other proteins and participating in the formation of the SNARE complex (5) (**Figure 2**). Thus, the switch between the closed and open conformations of syntaxin-1 is a switch for membrane fusion.

Munc18-1 is a member of the Sec1/Munc18 (SM) protein family. The domain 3a hinge-loop of Munc18-1 can catalyze the conversion of syntaxin-1A from a “closed,” fusion-incompetent conformation to an “open,” fusion-competent conformation. Munc13-1 is an isoform of Munc13 in major mammalian neurons. Munc13-1 contains four protein kinase C (PKC) homology domains: a calmodulin-binding domain (CaMb) and an extended tethering module called the MUN domain (26). Ca^2+^ and calmodulin (CaM) can bind to the corresponding domain of Munc13-1 to regulate the release of synaptic transmitters.

Munc18-1 interacts with syntaxin-1 in two modes. In mode I, free syntaxin-1 is protected by Munc18-1 into a closed, non-activated state, preventing the formation of the ternary SNARE complex and inhibiting neurotransmitter release (27). Mode II, also known as the N-terminal binding mode, in which Munc18-1 interacts with the conserved N-terminal domain of syntaxin-1a, is essential for the formation of the SNARE complex and the release of the neurotransmitter. Mode II interactions are regulated by Munc13, which catalyzes the transition of syntaxin-1 from a “closed” to an “open” conformation. One study found that mutations in the structural domain of rat Munc13-MUN abolished its stimulatory effect on SNARE complex assembly and membrane fusion, suggesting that the MUN domain underlies the catalytic action of Munc13-1, and that the MUN domain of Munc13 binds both syntaxin-1 and VAMP2 upon catalysis (28, 29). In addition, the mode II interaction is inhibited by the phosphorylation of syntaxin-1 at the Ser14 site (30).

UNC-13, a homolog of Munc13 in mammals, is a highly conserved syntaxin-binding protein that promotes the release of neurotransmitters. UNC-13 recruits synaptic vesicles for their release and also binds to syntaxin-1A for its open conformation, and the C2A domain of UNC-13L is identical to the corresponding domain of Munc13-1. Notably, inhalation of general anesthetics impairs the catalytic action of UNC-13 and promotes the closed conformation of syntaxin-1A (5).

### 3.3 Regulation of accessory proteins in binding of syntaxin-1 to other SNARE proteins

#### 3.3.1 SNARE is assembled into the correct pathway products under accessory protein regulation

The assembly of SNARE proteins into the pathway product requires the involvement of various accessory proteins, and without regulatory factors, SNAREs will incorrectly assemble into a variety of off-path products that do not mediate membrane fusion, limiting the speed and accuracy of SNARE assembly (31). For example, syntaxin-1 can form a homotetramer that cannot bind to SNAP-25 or VAMP2 (32). Syntaxin-1 and SNAP-25 can form not only a 1:1 pathway complex but also a 2:1 non-pathway complex. The 2:1 complex is more stable and its binding to VAMP2 is blocked by its own duplex syntaxin-1 (33). The antiparallel SNARE complex is also a misassembly of SNARE (31), and over 40% of complexes formed by mixing three synaptic SNAREs contain antiparallel helices (34).

Munc18-1 and Munc13-1 “template” SNARE complex assembly to ensure proper assembly orientation (34). Munc18-1 and syntaxin-1 binary complexes inhibit syntaxin-1 from participating in off-path assembly and generating erroneous SNARE complexes (35). Munc13-1, together with Munc18-1, opens syntaxin-1 (29) and also binds to the juxtamembrane region of VAMP2, templating the formation of the SNARE complex and making VAMP2 more accessible to the Munc18-1/syntaxin-1 complex (36). In the presence of SNAP-25, Munc18-1 releases syntaxin-1, leading to the complete assembly of the SNARE complex (37).

#### 3.3.2 The assembly of SNARE complexes regulated by accessory proteins is ordered and progressive

**Figure 3:**
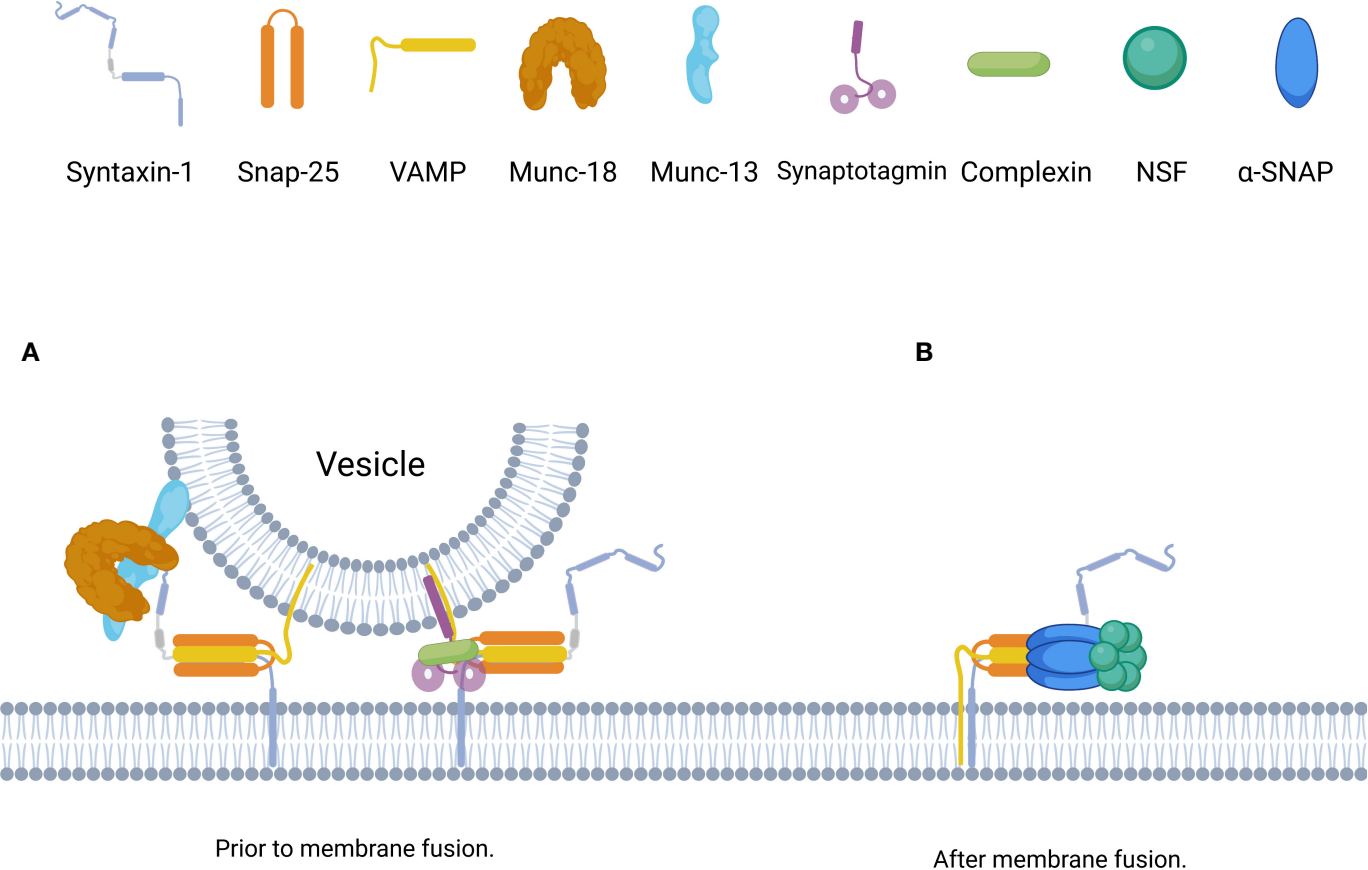
Schematic of the assembly and disassembly of the SNARE complex governed by distinct SNARE accessorys. **(A)** Prior to membrane fusion, the accessory proteins, including complexin, synaptotagmin, Munc18, and Munc13, form a basic interaction module with individual SNARE complexes. Munc18-1 and Munc13-1 bind to Syntaxin-1 to initiate and template the assembly of SNARE complexe, synaptotagmins and complexin bind to SNARE complexe to stabilize and lock SNARE complexe in intermediate states until Ca^2+^ binding to synaptotagmins. **(B)** After membrane fusion, trans-SNAREs are converted to cis-SNAREs which with both the Syntaxin-1 and VAMP residing in the same membrane,and can be disassembled by the joint action of α-SNAP and NSF with Mg-ATP.

During rapid exocytosis *in vivo*, SNARE complex formation is completed within 1 ms; during this period, a number of factors stabilize the SNARE complex in some intermediate states to ensure that the complexes bind together in a similarly ordered manner (12, 17) (**Figure 3**). By using surface force apparatus, magnetic tweezers, and electrophysiological measurements and by splitting VAMP2 peptides, the synaptic SNARE complex was found to exist in intermediate states during assembly *in vitro* (38). Non-synaptic SNARE complexes also bind together in a similar, stepwise manner (33).

The Ca^2+^ sensor synaptotagmin contains a family of transmembrane proteins with dual C2 domains that accelerate the fusion of synaptic vesicles and serves as the fusion clamp of mammalian neurons (39, 40). Synaptotagmin-1 is the primary Ca^2+^ sensor that induces synchronous brain release and consists of a short intravesicular N-terminal domain, a single TMR, a charged linker region, and two tandem C2 domains (C2A and C2B) (41). Synaptotagmin-1 participates in the SNARE-synaptotagmin-complex, which constitutes the priming and “locked” state of the vesicle: two synatotagmin-1 molecules bind simultaneously at the main interface of the complexin-SNARE complex (the C2B domain of synatotagmin-1 forms the main interface with the SNARE complex) and at the tripartite interface (the C2B domain of the other synatotagmin-1 forms the tripartite interface with complexin and the SNARE complex) (42). The tripartite interface simultaneously activates and “locks” the complex, keeping the energized trans-SNARE complex half-zippered and thereby preventing membrane fusion. Ca^2+^-binding neutralizes the charge of the C2 domains to facilitate membrane penetration by synaptotagmin-1, which unlocks the tripartite interface and allows the trans-SNARE complex to fully zipper, thereby triggering fusion. In addition, membrane penetration by synaptotagmin-1 may also directly facilitate fusion (43). Moreover, synaptotagmin-1 helps to prevent NSF/α-SNAP-mediated decomposition of trans-SNARE complex (44). Synaptotagmin-2 is highly similar to synaptotagmin-1 in mediating the release of rapidly synchronized neurotransmitters (37).

Complexin, also known as synaphin, mediates the localization of proteins and the binding of synaptic vesicles, facilitates the fusion of highly curved membranes, and regulates SNARE complexes and neurotransmission. The central α-helix of the complex can also form part of the tripartite interface of the “primed, locked” synaptotagmin-complex-SNARE complex, which enhances the rapid release of neurotransmitters in response to stimulation by calcium signals (42, 45). Complex proteins and α-SNAP compete to bind SNARE, and the binding of complexin to the SNARE complex promotes the stability of the primed metastable SNARE complex and facilitates the assembly of the SNARE complex (42). However, complexin inhibits fusion: a liposome experiment showed that complexin keeps trans-SNARE complexes in an intermediate state with fused outer liposome membranes and distinct inner membranes, which can be alleviated by calcium and synaptotagmin (46). The role of complex proteins is different in mammals and invertebrates. Complex proteins can effectively inhibit spontaneous release in invertebrates, but the evidence in mammals is not clear (47).

#### 3.3.3 SNARE complex assembly efficiency is regulated

In addition to the synaptotagmin and complexin mentioned above, a number of protein factors can accelerate or inhibit the process of SNARE complex formation and regulate vesicle docking and fusion directly or indirectly, including tomosyn, proline-rich transmembrane protein 2 (PRRT2), Sept5, Syntaphilin, α-SNAP and NSF.

Tomosyn is a syntaxin-1A-binding protein divided into tomosyn1 and tomosyn2, which inhibit the initiation steps of neurosecretory cells and neurons. The C-terminus of all isoforms of tomosyn contains a C-terminal domain homologous to VAMP2, which forms a tight complex with t-SNAREs and prevents VAMP2 from entering the SNARE complex (48). VAMP2 cannot displace tomosyn from the SNARE core complex, but the inhibitory effect of tomosyn-1 on the SNARE complex can be antagonized by NSF/α-SNAP, indicating that the tomosyn/SNARE complex generated by physiological conditions is a “dead-end” complex. Tomosyn was found to significantly reduce neurosecretory cell and neuronal exocytosis, and disruption of the tomosyn homolog Tom1 resulted in enhanced neurotransmitter release (19, 37).

PRRT2, which is highly enriched in the presynaptic plasma membrane and also present on small synaptic vesicles, consists of a proline-rich domain within the unstructured N-terminal cytoplasmic region, two transmembrane domains linked by a cytosolic loop, and an extracellular C-terminal tail (49). PRRT2 binds SNARE proteins (predominantly Syntaxin-1A and SNAP-25) and synaptotagmin-1/-2 (50). Additional research has shown that PRRT2 blocks the assembly of trans-SNARE complexes *in vitro* and inhibits liposome docking and synaptotagmin-dependent proteoliposome fusion (51). Mutations in PRRT2 result in haploinsufficiency or loss of function of the protein, increased assembly of the SNARE complex, increased docking of vesicles, and changes in cell- and region-specific neurotransmission and network activity (37). These findings demonstrate that PRRT2 is a negative regulator of vesicle initiation and fusion.

Sept5 (previously called CDCrel-1) is associated with the components of the membrane fusion apparatus, and contains a GTP-binding motif as well as a predicted coiled-coil domain. Syntaxin binds to both ends of Sept5, including the N-terminal portion encompassing the GTP-binding region (52). Sept5 inhibits regulated exocytosis when overexpressed in HIT-T15 cells. Furthermore, platelet secretion assays using platelets from Sept5NULL mice showed an enhanced platelet secretion response (53). Sept5 binds to Syntaxin in the SNARE complex, this interaction is occluded by the binding of α-SNAP (52), and displacement of Sept5 by α-SNAP would then allow binding of NSF and the subsequent dissociation of the cis 7S complexes (cis-SNARE complexes) on the vesicle and membrane (52).

Syntaphilin is a brain-enriched protein that includes 13 repeats of proline-rich motifs (PPXXPP, PXXP, or PXP) and numerous consensus protein phosphorylation sites (11 sites for PKC, three sites for cAMP-dependent protein kinase (PKA)/cGMP-dependent protein kinase (PKG), and eight sites for CaMKII), suggesting that it may be the target for modulation of synaptic transmission through signal transduction pathways at nerve terminals (54). Transient overexpression of syntaphilin in cultured hippocampal neurons or introduction of syntaphilin coiled-coil domain (CC) into presynaptic superior cervical ganglion neurons (SCGNs) in culture significantly reduces neurotransmitter release (54, 55). By binding to the H3 CC of syntaxin-1, syntaphilin prevents syntaxin-1 from interacting with SNAP-25 and blocks SNARE complex formation by absorbing the free syntaxin-1 at nerve terminals (55).

NSF is a soluble cytosolic hexamer protein and SNAPs are a family of cytoplasmic proteins including α, β, and γ homologs (56). α-SNAP activates NSF, and three α-SNAPs bind to the SNARE complex and the NSF hexamer binds to the periphery of the SNAP/SNARE complex to form a 20S complex. In the presence of ATP, NSF hydrolyzes ATP to release energy, allowing the SNARE complex to depolymerize for reuse (56). α-SNAP and NSF promote the formation of the trans-SNARE complex, regulate the size of the vesicle pool that can be released prior to fusion after docking, and depolymerize the cis-SNARE complex after membrane fusion (56) (**Figure 3**).

## 4 Non-accessory protein regulation of syntaxin-1

### 4.1 Syntaxin-1-related phosphorylation

The reversible phosphorylation of proteins is a general control mechanism that regulates almost all aspects of cell life, including the protein interactions within the exocytosis machinery (57). Cyclin-dependent kinase 5 (Cdk5), Ca^2+^-calmodulin-dependent kinase II (CaMKII), Serine/threonine kinase casein kinase 2α (CK2α), Death-associated protein kinase (DAPK), cAMP-dependent protein kinase (PKA), and Protein kinase C (PKC) activation in the presynaptic terminus have been shown to correlate with the protein binding of syntaxin-1.

Cdk5, a proline-directed serine/threonine kinase, is predominantly expressed in the nervous system. Cdk5 activated by p35 was found to bind to and phosphorylate Munc-18. The phosphorylation site on Munc-18a was identified as Thr574 (58), and the phosphorylated Munc-18 showed significantly reduced affinity for syntaxin-1A. Cdk5 can also bind to syntaxin-1A, and a complex of p35, Cdk5, Munc-18, and syntaxin 1A can be formed in the absence of ATP and promptly disassembled upon the addition of ATP (59). Cdk5/p35 phosphorylates Sept5 *in vitro* and *in vivo*, and S327 of Sept5 is a phosphorylation site. A serine (S)-to-alanine (A) 327 mutant of Sept5 bound syntaxin more efficiently than the wild type. Additionally, the phosphorylation of Sept5 by Cdk5/p35 decreased its binding to syntaxin-1. Moreover, the mutant non-phosphorylated Sept5 potentiated exocytosis further than the wild type when expressed in PC12 cells (58). Another study showed that phosphorylation of adult-type Sept5 (Sept5_v1) at Ser17 by Cdk5-p35 reduced the binding of Sept5_v1 to syntaxin-1 (60).

CaMKII is a multimeric enzyme that can affect the assembly of the SNARE complex and includes α, β, γ, and δ isoforms (61). In the resting state, the catalytic region of CaMKII is covered by a self-inhibitory region and is inactive. Certain stimuli cause Ca^2+^/CaM to bind to the regulatory region of CaMKII, exposing the catalytic region of CaMKII and preventing the self-inhibitory region from inhibiting the activation site (62). In the presence of Ca^2+^ and ATP, phosphorylated CaMKII binds reversibly and specifically to the linker region of syntaxin-1 (63), and the bound complex promotes the assembly of SNAREs. The CaMKII-binding fragment of syntaxin-1 reduces the frequency of exocytosis, which may result from competitive inhibition of the interaction of CaMKII with syntaxin-1. CaMKII and Munc-18 alternatively bind to syntaxin-1, and CaMKII binding to the open conformation of syntaxin-1 appears to maintain the openness of syntaxin-1 (63). Notably, CaM/CaMKII inhibitors appear to repress syntaxin-1a expression (64).

CK2α is ubiquitously expressed in eukaryotic cells (31) and maintains neuronal presynaptic stability, specifically catalyzing the phosphorylation of Ser14 in syntaxin-1 (65), and the phosphorylation site in syntaxin-1 Ser14 regulates the N-terminal interaction with Munc18-1, which is the mode II interaction mentioned above. Reduction in CK2α-dependent syntaxin-1 phosphorylation can lead to an increase in the extracellular secretion of persistent synaptic vesicles. Furthermore, CK2α phosphorylation of syntaxin-1 regulates the apparent number of releasable synaptic vesicles (66).

DAPK is a 160-kDa, calmodulin-regulated, and cytoskeleton-associated serine/threonine kinase composed of a kinase domain, a CaM regulatory domain, eight consecutive ankyrin repeats, two putative nucleotide binding domains, a cytoskeletal/ras of complex proteins (ROC) domain, and a death domain (67, 68). DAPK undergoes activation in response to various death stimuli, and they have been associated with an increase in DAPK catalytic activity. DAPK phosphorylates syntaxin-1A at Ser188 in a Ca^2+^-dependent manner, and the phosphorylation event has been shown to reduce the binding of syntaxin to Munc18-1 (68).

Protein phosphorylation by PKC and PKA have been implicated in the control of neurotransmitter release, and the effects of PKC and PKA on the activation of syntaxin-1 may be indirect. SNAP-25 and syntaphilin can be phosphorylated by PKA both *in vitro* and *in vivo*. PKA catalyzes the phosphorylation of Thr-138 in SNAP-25, and overexpression of the Thr-138 mutant SNAP-25a eliminated the effects of PKA inhibitors on the vesicle-priming process (69). Additionally, phosphorylation of syntaphilin by PKA inhibits the binding of syntaphilin to syntaxin-1A. Mutation of the dominant phosphorylation site serine 43 to aspartic acid (mimicking 100% of phosphorylation) can annul the inhibitory effect of syntaphilin on Ca^2+^-dependent exocytosis in PC12 cells (55).

Munc18a can be phosphorylated by PKC *in vitro* on Ser-306 and Ser-313, and mutations of both phosphorylation sites to glutamate reduces its affinity for syntaxin (70). One study found that SNAP-25 is phosphorylated at the residue serine-187 by PKC, and mutant mice lacking the phosphorylation of SNAP-25 serine-187 exhibit reduced release probability and enhanced presynaptic short-term plasticity (71).

### 4.2 Syntaxin-1-related lipid regulation

In addition to proteins, lipid molecules also function as significant active regulators in SNARE-mediated membrane fusion from two aspects: protein assembly and membrane curvature (81). Different lipid components, including phosphatidic acid (PA), phosphatidylinositol phosphates (PIPs), and cholesterol, affect syntaxin-1A activation-related processes such as oligomerization and SNARE assembly (9, 73).

PA, a fusogenic lipid, directly binds to a polybasic juxtamembrane region within syntaxin-1A (73, 74). In one study, rat brain slices were exposed to 5 µM PA for 2 h, and their glutamate-releasing ability on KCl stimulation was assessed. The results indicated a near-threefold increase in the potency and efficacy of glutamate release at the early timepoint (10 min), which plateaued subsequently (74). Syntaxin-1A mutations that progressively reduce lipid binding could result in a progressive loss in cell secretion (73). On the other hand, overexpression of the PA-generating enzyme phospholipase D1 rescued the mutant from secretion defects (73).

PIPs also play a key role in syntaxin-1 clustering. Phosphatidylinositol-4,5-bisphosphate (PIP2) is concentrated at the junction of secretory vesicles and is associated with syntaxin-1. The interaction between PIP2 and syntaxin-1 allows syntaxin-1 to cluster in PC12 cells. Calcium can also act as a charge bridge connecting multiple syntaxin-1 and PIP2 complexes and can allow PIP2 junctions to form a larger domain to regulate the size of syntaxin clusters (75). One study suggests that PIP2 regulates the phosphorylation of the syntaxin N-terminus by modulating both its position and local structure (76). Syntaxin-1/PIP2 domains represent the preferred binding sites for synaptotagmin-1, synaptotagmin-1 interacts with the polybasic linker region of syntaxin-1A independent of Ca(2+) through PIP2 (77).

Phosphatidylinositol-3,4,5-bisphosphate (PIP3) also promotes syntaxin-1 clusters. The interaction of PIP3 with syntaxin-1 is required for syntaxin-1 clusters and neurotransmitter release in NMJs (78). The PIP3 binding-deficient mutant syntaxin-1AKARRAA-mEos2 in *Drosophila* larvae NMJs was found to show smaller clusters than wild-type syntaxin-1A-mEos2. Syntaxin-1AKARRAA-mEos2 shows higher mobility in NMJs than wild-type syntaxin-1A-mEos2 and does not vary with thermogenic stimulation (20).

Cholesterol controls the stability of different protein assemblies on the plasma membrane and also regulates syntaxin-1 clustering. Cholesterol is thought to be a key regulator of syntaxin-1 cluster stability, and its depletion at the plasma membrane markedly reduced syntaxin-1 clustering at the plasma membrane of PC12 cells and pancreatic β-cells (79). In addition, changes in cholesterol levels affect the thickness of the membrane, which in turn affects the local hydrophobic mismatch (wherein the length of the transmembrane domain region of a membrane protein differs from the hydrophobic thickness of the membrane, affecting the conformation, folding, activity, and aggregation of the protein), regulating the clustering of syntaxin-1 (80).

### 4.3 Syntaxin-1-related exocytosis as a target for certain drugs and toxins

In addition to the endogenous factors mentioned above, certain exogenous factors can have an effect on syntaxin-regulated exocytosis, including botulinum neurotoxins (BoNTs), antiepileptic drugs, anesthetic drugs and antidepressant drugs.

BoNTs are metalloproteinases that can be endocytosed into neuron nerve terminals and translocation of the light chain across the membrane, and then cleaves SNARE proteins (37, 82). However, the hydrolysis of SNARE proteins caused by BoNTs leads to the failure of neurotransmitter transmission and causes typically fatal flaccid paralysis (83). Depending on the serotype, BoNTs are classified into seven types, named from BoNT/A to BONT/G (82). BoNT/A and E cleave SNAP-25, BoNT/B, BoNT/D, BoNT/F and BoNT/G cleave VAMP-1/2, whereas BoNT/C cleaves syntaxin-1/2 and, albeit with lower efficiency, SNAP-25 (82). One study found that truncated SNAP-25 caused by BoNT/A increased the flickering of fusion pores and inhibited exocytosis (72), suggesting that BoNT/A is likely to destabilise the trans-SNARE complex and fusion pore.

Syntaxin-1-associated exocytosis mechanisms are likely to be novel targeting mechanisms for the antiepileptic actions of antiepileptic drugs (84) such as carbamazepine (CBZ), valproate (VPA), and zonisamide (ZNS). CBZ and ZNS increase Syntaxin-regulated basal neurotransmitter release and Ca^2+^-evoked release, and their stimulatory actions are predominantly inhibited by inhibitors of N-type voltage-sensitive calcium channels (VSCC) and syntaxin. In addition, CBZ and ZNS reduce VAMP-regulated K^+^-evoked release, an action predominantly inhibited by inhibitors of P-type VSCCs and synaptobrevin (85). Thus, the mechanisms underlying the antiepileptic action of CBZ, VPA, and ZNS may be mediated by a combination of effects resulting from enhanced basal release of inhibitory neurotransmitter without affecting basal glutamate release, and reduced neurotransmitter release at the event of neuronal excitation channels (i.e., during seizures) (85).

Syntaxin-1-associated exocytosis mechanisms are likely to be one of the targeting mechanisms of anesthetic drugs to produce anesthetic action (86). Isoflurane (a volatile anesthetic) decreases transmitter release by reducing the release probability (the chance of a vesicle undergoing exocytosis after an action potential). SyxH3-C, a 14-amino acid deletion mutant of syntaxin-1A, renders animals resistant to isoflurane, but syxKARRAA, a strain with two amino acid substitutions in syntaxin-1A, hypersensitizes animals to the drug (87). Intravenous anesthetics such as propofol and etomidate can limit the migration of syntaxin-1A, while their analogues increase the migration of syntaxin-1A. Removal of the interaction of syntaxin-1 with SNAP25 can eliminate the migration restrictions of syntaxin-1A by propofol, suggesting that propofol interferes with the establishment of the release site, affecting a step in the formation of the SNARE complex and resulting in a nonfunctional syntaxin-1A cluster that disables cell fusion (88, 89). Notably, propofol significantly reduces the expression and phosphorylation levels of CaMKII (63).

Syntaxin-1-related glutamate neurotransmission has also been implicated in the action of stress and in antidepressant mechanisms (90). Chronic treatment with three antidepressants with different primary mechanisms (fluoxetine, reboxetine, and desipramine) was shown to selectively reduce the expression levels of synaptobrevin, syntaxin-1, and SNAP-25, and markedly reduce the depolarization-evoked release of glutamate stimulated by 15 or 25 mM KCl, but not the release of GABA (91). Moreover, another study found that Thr 286 autophosphorylation of αCaMKII associated with synaptic membranes was reduced by 70%-80%, the syntaxin 1/αCaMkII interaction was reduced by 60%-70%, and the syntaxin 1/Munc-18 interaction was augmented by up to 200% in drug-treated rats (90, 91).

## 5 Syntaxin-1 mutations in diseases

Genetic alterations in Syntaxin-1 can cause NE disruption, leading to a variety of metabolic and neurological symptoms. We have summarized the current state of research on syntaxin-1 gene-related disorders in the paragraphs below.

Deletion of syntaxin-1A and B in mice is associated with embryonic lethality and leads to gross morphological abnormalities and neuronal cell loss both *in vivo* and *in vitro*, resulting in a complete loss of fusion function of synaptic vesicles and severely disrupting vesicle docking (92).

Syntaxin-1A knockout (KO) mice are viable and fertile. These mice display normal spontaneous and evoked release, basal transmission and paired-pulse ratio, but impaired long-term potentiation (LTP, a cellular model of memory) (93, 94). Correspondingly, heterozygous and homozygous syntaxin-1A KO mice show some behavioral abnormalities in social and object recognition, fear conditioning, and acoustic startle response (95). Syntaxin-1A KO in mice has been reported to result in a severe defect in extracellular insulin secretion in mice and a defect in the hypothalamic-pituitary-adrenal axis for corticosterone and catecholamine release (96, 97). In patients with type 2 diabetes, syntaxin-1A levels in pancreatic beta cells are severely reduced, suggesting that the absence of syntaxin-1 may lead to impaired insulin secretion (98). Moreover, *STX1A* (encoding Syntaxin-1A) is one of approximately 28 genes within a commonly deleted region in Williams Syndrome (or Williams-Beuren Syndrome, OMIM 194050), a multi-system developmental syndrome with features such as connective tissue abnormalities, cardiovascular disease, mild intellectual disability, delayed motor development, unique social and personality characteristics, and growth and endocrine abnormalities (99, 100).

In contrast, syntaxin-1B KO mice show severely disrupted motor coordination, impaired brain development, and death within two weeks of age (101). Heterozygous mutations in syntaxin-1B cause a wide spectrum of epileptic disorders from genetic generalized epilepsies (GGE) and genetic epilepsies with febrile seizures plus (GEFS+) to more severe developmental and epileptic encephalopathies (DEE) that feature early-onset intractable seizures, cognitive regression, and neurological deficits (102). However, syntaxin-1B heterozygous mice show normal growth, survival, and brain structure, and have no effect on action potential-evoked, sucrose-evoked, or spontaneous release at hippocampal or neuromuscular synapses (103). In contrast, zebrafish larvae with 50% knockdown of syntaxin-1B lacked normal touch response and exhibited paroxysmal movements and jerks (104).

## 6 Summary

**Figure 4:**
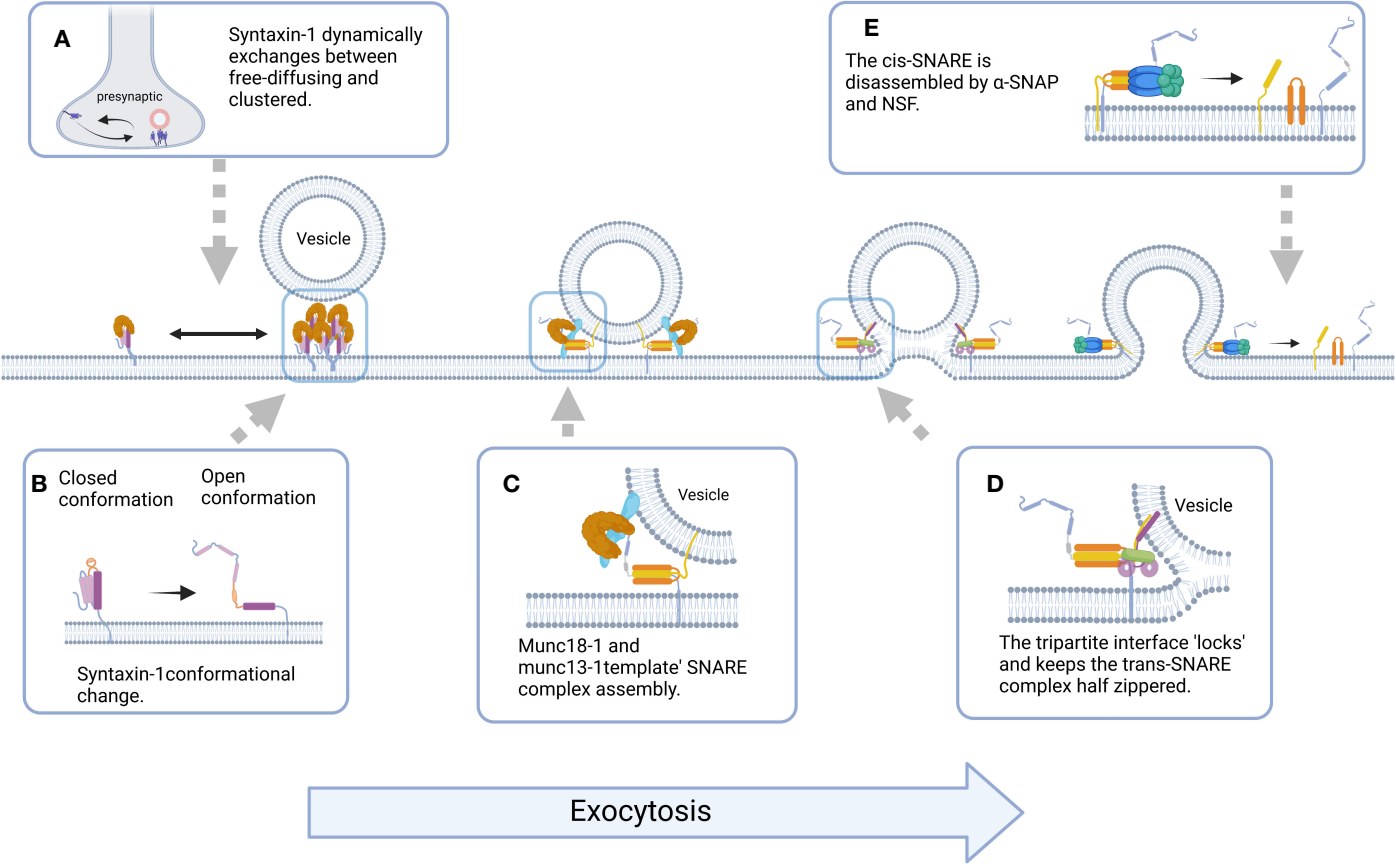
Schematic representation of the molecular events underlying Syntaxin-1-mediated exocytosis. **(A)** Syntaxin-1 molecules constantly switches between freely diffusing (mobile) and clustered (immobile) states at the plasma membrane, and Syntaxin-1 clusters could act as docking and fusion sites for secretory vesicles. **(B)** The switch between the closed and open conformation of Syntaxin-1 is a switch for membrane fusion. **(C)** Munc18-1 and Munc13-1 ‘template’ SNARE complex to ensure assembly of SNAREs proteins into pathway product. **(D)** The tripartite SNARE-complexin-synaptotagmin-1 complex has to be unlocked for triggered fusion to commence, which contributes to synchronizing evoked release on the sub-millisecond timescale. **(E)** Binding of α-SNAP and NSF to cis-SNARE leads to the disassembly of SNAREs and the release of proteins involved in vesicle fusion. (All figures created with Biorender.com.).

Syntaxin-1 mediates the docking and fusion of synaptic vesicles and presynaptic membranes by facilitating the assembly of the SNARE complex, and a growing body of evidence indicates that regulation of syntaxin-1 is an important event for proper synaptic function. The changes in the open and closed conformations of syntaxin-1, nanoclusters formed by syntaxin-1, and the orderly, rapid, and accurate assembly of SNARE complexes involving syntaxin-1 are the main mechanisms of action of multiple cofactors (**Figure 4**), and a variety of non-accessory protein mechanisms such as lipid binding, phosphorylation regulation, and drug toxin action are also involved in the regulation of syntaxin-1 (**Table 1**). Therefore, the key factors regulating the physiological functions of syntaxin-1 and their roles have become a research “hotspot” in subcellular proteomics.

**Table 1:** Factors regulating the function of syntaxin-1.

Factor	Related Events	Effect and mechanism
SNAP-25	Syntaxin-1 clustering (21, 22). Assembly of SNARE complexes (21, 22).	SNAP-25 and VAMP form SNARE complex with Syntaxin-1 to promote the cluster of Syntaxin-1 on the plasma membrane. Blocking the formation of the SNARE complex increases the mobility of Syntaxin-1 and reduces the size of Syntaxin-1 nanoclusters (21, 22).
VAMP	Syntaxin-1 clustering (21). Assembly of SNARE complexes (21).	SNAP-25 and VAMP form SNARE complex with Syntaxin-1 to promote the cluster of Syntaxin-1 on the plasma membrane (16). Tetanus toxin (cleaves VAMP) prevented the formation of the SNARE complex, leading to an increase in the mobility of Syntaxin-1 and a decrease in the size of the Syntaxin-1 nanocluster on neuromuscular junction (NMJ) (21).
Syntaxin-1	Syntaxin-1 clustering (24, 25).	The TMR of Syntaxin-1 can form clusters individually and the interaction between Syntaxin-1 allows Syntaxin-1 to loosely cluster at the plasma membrane and maintain a dynamic balance of free and clustered subpopulations (24, 25).
Munc18-1	Syntaxin-1 clustering (23). Conformational change of Syntaxin-1 (27–29). Assembly of SNARE complexes (34, 35).	Munc18-1 affects Syntaxin-1 mobility by mediating the assembly of the SNARE complex (23). Mode I: Munc18-1 protects Syntaxin-1 into a closed conformation (27). Mode II: Munc18-1 Binds to the N-terminal end of Syntaxin-1, opens Syntaxin-1 and regulates SNARE complex formation (28, 29). Munc18-1 complexed with Syntaxin-1 inhibits the production of Syntaxin-1 error SNARE complexes (34, 35).
Munc-13/UNC-13	Conformational change of Syntaxin-1 (5, 28, 29). Assembly of SNARE complexes (36).	Munc13 catalyzes the transition of Syntaxin-1 from a “closed” to an “open” conformation, and the MUN domain of Munc13 binds to both Syntaxin-1 and VAMP2 upon catalysis (5, 28, 29). Munc13-1 binds to the membrane proximal region of VAMP2, making VAMP2 more accessible to Munc18-1/Syntaxin-1 templated SNARE formation (36).
Synaptotagmins	Assembly of SNARE complexes (42, 44).	Synaptotagmins promote complete closure of trans-SNARE to trigger fusion (42) and prevent NSF/α-SNAP decomposes trans-SNARE (44). Synaptotagmins participate in the formation of SNARE-synaptotagmin-complexin (42).
Complexin	Assembly of SNARE complexes (42, 45, 46).	Complexin participates in the formation of SNARE-synaptotagmin-complexin (42, 45). Complex proteins and α-SNAP compete to bind SNARE, and binding of complexin to the SNARE complex promotes the stability of the primed metastable SNARE complex and facilitates the assembly of the SNARE complex (42). Complexin maintains the intermediate state of trans-SNARE complex in semi fusion (46).
Tomosyn	Assembly of SNARE complexes (19, 37).	Tomosyn binds t-SNAREs to form a tight complex, preventing VAMP-2 from entering the SNARE complex (19, 37).
PRRT2	Assembly of SNARE complexes (50, 51).	PRRT2 binds SNARE proteins (predominantly Syntaxin-1A and SNAP-25) and synaptotagmin-1/-2 (50), PRRT2 blocks trans-SNARE complex assembly (51).
Sept5	Assembly of SNARE complexes (52, 53).	Sept5 inhibited regulated exocytosis (53). Displacement of Sept5 by α-SNAP would allow binding of NSF, and the subsequent dissociation of the cis-SNARE complexes (52).
Syntaphilin	Assembly of SNARE complexes (55).	By binding to the H3 CC of syntaxin-1, syntaphilin prevents syntaxin-1 from interacting with SNAP-25 and blocks SNARE complex formation by absorbing free syntaxin-1 at nerve terminals (55).
NSF,α-SNAP	Assembly of SNARE complexes (56).	α-SNAP and NSF promote the formation of trans-SNARE (56) and depolymerize cis-SNARE after membrane fusion (56).
CDK5	Syntaxin-1 related phosphorylation (58, 59). Binding of Sytaxin-1 accessory proteins (58).	Cdk5 binds to and phosphorylates Munc-18, and the phosphorylated Munc-18 has a significantly reduced affinity for Syntaxin 1A (58). Cdk5 can bind to Syntaxin-1A (59). Cdk5 can phosphorylate Sept5, and phosphorylation of Sept5 by Cdk5/p35 decreased its binding to Syntaxin-1 (58).
CaMKII	Syntaxin-1 related phosphorylation (62, 63). Assembly of SNARE complexes (63). Conformational change of Syntaxin-1 (63).	Phosphorylated CaMKII binds Syntaxin-1, and the CaMKII-Syntaxin-1 complex promotes SNARE assembly (63). CaMKII and Munc-18 alternatively bind to Syntaxin-1, and CaMKII binding to the open conformation Syntaxin-1 appears to maintain the openness of Syntaxin-1 (63). CaM/CaMKII inhibitors appear to repress Syntaxin-1a expression (64).
CK2α	Syntaxin-1 related phosphorylation (65). Conformational change of Syntaxin-1 (65).	CK2α catalyzes the phosphorylation of Ser14 in Syntaxin-1 and inhibits mode II interactions (65).
DAPK	Syntaxin-1 related phosphorylation (68). Binding of Sytaxin-1 accessory proteins (68).	DAPK phosphorylates Syntaxin1A at Ser188 in a Ca^2+^ dependent manner, and the phosphorylation event has been shown to reduce the binding of Syntaxin to Munc18-1 (68).
PKA	Syntaxin-1 related phosphorylation (55, 69). Binding of Sytaxin-1 accessory proteins (55). Assembly of SNARE complexes (69).	PKA catalyzing the phosphorylation of Thr-138 in SNAP-25 (69), phosphorylation of syntaphilin by PKA inhibits the binding of syntaphilin to Syntaxin-1A (55).
PKC	Syntaxin-1 related phosphorylation (70, 71). Binding of Sytaxin-1 accessory proteins (70). Assembly of SNARE complexes (71).	Munc18a can be phosphorylated by PKC *in vitro* on Ser-306 and Ser-313, and Mutation of both phosphorylation sites to glutamate reduces its affinity for Syntaxin (70), and SNAP-25 is phosphorylated at the residue serine-187 by PKC (71).
Phosphatidic acid	Syntaxin-1 related lipid regulation (73, 74).	Phosphatidic acid directly binds to a polybasic juxtamembrane region within syntaxin-1A (73, 74). Syntaxin-1A mutations, that progressively reduce lipid binding, could result in a progressive loss in cell secretion (73).
PIP2	Syntaxin-1 related lipid regulation (75–77). Syntaxin-1 clustering (75, 77).	Phosphatidylinositol phosphates concentrates at the fusion site and interacts with Syntaxin-1 to promote Syntaxin-1 cluster (75). PIP2 regulate the phosphorylation of syntaxin N-terminus by modulating both its position and local structure (76). Syntaxin-1/PIP2 domains represent the preferred binding sites for synaptotagmin-1 (77).
PIP3	Syntaxin-1 related lipid regulation (20, 78). Syntaxin-1 clustering (20, 78).	PIP3 also promotes Syntaxin-1 clusters (78), PIP3 binding-deficient mutant Syntaxin-1AKARRAA-mEos2 in Drosophila larvae NMJs has smaller clusters,and has higher mobility in NMJs than wild-type Syntaxin-1A-mEos2 (20).
Cholesterol	Syntaxin-1 related lipid regulation (79, 80). Syntaxin-1 clustering (79, 80).	Cholesterol controls the stability of protein assemblies at the plasma membrane (79) and regulates Syntaxin-1 cluster at the plasma membrane (80).
BoNTs	Cleavage of SNARE proteins (37, 82, 83).	BoNTs cleave neuronal SNARE proteins, and causes typically fatal flaccid paralysis (37, 83). BoNT/A and E cleave SNAP-25, BoNT/B, BoNT/D, BoNT/F and BoNT/G cleave VAMP-1/2, whereas BoNT/C cleaves syntaxin-1/2 and, albeit with lower efficiency, SNAP-25 (82).
Antiepileptic drug	Syntaxin-1-associated exocytosis (85).	CBZ and ZNS increased Syntaxin-regulated basal neurotransmitter release and Ca^2+^-evoked neurotransmitter release, these stimulatory actions were predominantly inhibited by inhibitors of N-type VSCC (voltage-sensitive calcium channels) and syntaxin. In addition, CBZ and ZNS reduced VAMP-regulated, K^+^-evoked release, an action predominantly inhibited by inhibitors of P-type VSCCs and VAMP (85).
Anesthetic drug	Syntaxin-1 clustering (88, 89). Syntaxin-1-associated exocytosis (87–89).	SyxH3-C, a 14-amino acid deletion mutant of Syntaxin-1A, renders animals resistant to isoflurane, but syxKARRAA, a strain with two amino acid substitutions of Syntaxin-1A, hypersensitizes animals to the drug (87). propofol and etomidate can limit the migration of Syntaxin-1A, and their analogues increase the migration of Syntaxin-1A. Removal of the interaction of Syntaxin-1 with SNAP25 eliminated the migration restriction of Syntaxin-1A by propofol (88, 89).
Antidepressant drug	Expression of SNARE proteins (91). Binding of Sytaxin-1 accessory proteins (90, 91).	Chronic treatment with three antidepressants with different primary mechanisms (fluoxetine, reboxetine, and desipramine) selectively reduced the expression levels of synaptobrevin, Syntaxin-1, SNAP-25 (91). The interaction Syntaxin-1/αCaMkII was reduced by 60-70% and the interaction syntaxin 1/Munc-18 was augmented up to 200% in drug-treated rats (90, 91).

In the course of the study, altered syntaxin-1 function was also shown to cause NE disorders, leading to various metabolic and neurological diseases. However, NE toxicities may also produce clinical therapeutic effects, such as antiepileptic and anesthetic effects. Syntaxin-1 is widely distributed in the nervous and endocrine systems and is a key protein in studies of various neurological and psychiatric diseases and drugs. However, research on the exact mechanism of action of syntaxin-1 as a drug target is limited, indicating new directions for future studies.

## Author contributions

XY, JZ: original draft preparation. XY: software, figure, and table. XG, WT and QZ: revising the manuscript critically. QZ, JG and JZ: reviewing and editing. All authors contributed to the article and approved the submitted version.
